# Factors affecting marginal integrity of class II bulk-fill composite resin restorations

**DOI:** 10.15171/joddd.2017.019

**Published:** 2017-06-21

**Authors:** Siavash Savadi Oskoee, Mahmoud Bahari, Elmira Jafari Navimipour, Amir Ahmad Ajami, Negar Ghiasvand, Ayda Savadi Oskoee

**Affiliations:** ^1^Department of Operative Dentistry, Faculty of Dentistry, Tabriz University of Medical Sciences, Tabriz, Iran; ^2^Dental and Periodontal Research Center, Tabriz University of Medical Sciences, Tabriz, Iran; ^3^Department of Operative Dentistry, Faculty of Dentistry, Urmia University of Medical Sciences, Urmia, Iran

**Keywords:** Composite resin, bulk fill, curing light, marginal gap, silorane, methacrylate

## Abstract

***Background.*** Bulk-fill composite resins are a new type of resin-based composite resins, claimed to have the capacity to be placed in thick layers, up to 4 mm. This study was carried out to evaluate factors affecting gap formation in Cl II cavities restored using the bulk-fill technique.

***Methods.*** A total of 60 third molars were used in this study. Two Cl II cavities were prepared in each tooth, one on the mesial aspect 1 mm coronal to the CEJ and one on the distal aspect 1 mm apical to the CEJ. The teeth were divided into 4 groups: A: The cavities were restored using the bulk-fill technique with Filtek P90 composite resin and its adhesive system and light-cured with quartz tungsten halogen (QTH) light-curing unit. B: The cavities were restored similar to that in group A but light-cured with an LED light-curing unit. C: The cavities were restored using the bulk-fill technique with X-tra Fil composite resin and Clearfil SE Bond adhesive system and light-cured with a QTH curing unit. D: The cavities were restored similar to that in group C but light-cured with an LED light-curing unit. The gaps were examined under a stereomicroscope at ×60. Data were analyzed with General Linear Model test. In cases of statistical significance (P<0.05), post hoc Bonferroni test was used for further analyses.

***Results.*** The light-curing unit type had no effect on gap formation. However, the results were significant in relation to the composite resin type and margin location (P<0.001). The cumulative effects of light-curing unit*gingival margin and light-curing unit*composite resin type were not significant; however, the cumulative effect of composite rein type*gingival margin was significant (P=0.04)

***Conclusion.*** X-tra Fil composite exhibited smaller gaps compared with Filtek P90 composite with both light-curing units. Both composite resins exhibited smaller gaps at enamel margins.

## Introduction

Despite great advances in the field of composite resin technology and extension of its applications in restorative dentistry, they still have disadvantages, including high wear rate, low strength, technique sensitivity and more importantly, polymerization shrinkage that gives rise to gap formation at the restorative material‒cavity wall interface, leading to microleakage due to the internal and interfacial stresses it creates.^1^ Microleakage leads to recurrent caries, postoperative sensitivity, marginal discoloration and loss of the restoration.^2,3^ Therefore, a large number of studies have been undertaken on techniques that result in a decrease in polymerization shrinkage, including placement of composite resins using the incremental technique, use of materials with low elastic modulus to absorb stresses and use of techniques to decrease the C-factor.^4-7^ Since these techniques are time-consuming and increase the chair time, resin-based composite (RBC) manufacturers have made significant developments to reduce the shrinkage stress generated on light irradiation and today dentistry boasts of RBC filler technology that encompasses nanotechnology,^8^ polymerization modulators technology^9^ and non-methacrylate-based monomeric resin formulations.^10^

One of these non-methacrylate-based monomeric resin formulations is silorane-based composite resins which are a combination of siloxane and oxirane under a cationic ring-opening polymerization.^11,12^ The silorane-based composite resins undergo volumetric shrinkage of 0.99%,^13^ which is significantly less than that of methacrylate-based composite resins (2.9‒7.1%).^14^ Compared to methacrylate-based composite resins, in silorane-based composite resins, polymerization stresses are compensated by opening an oxirane ring element.^15^ Opening of oxirane rings during polymerization compensates the volumetric decrease resulting from packing of the monomers.^16^ In relation to silorane-based composite resins, evaluation of the cohesive bond of the incremental layers has shown that the layers of this type of composite resin exhibit poorer cohesive bond properties compared to dimethacrylate composite resins due to the absence of oxygen-inhibited layer and a different polymerization mechanism. It is advisable to use the bulk-fill technique to place such composite resins in the cavity.^17^

Other products are bulk-fill methacrylate-based composite resins using polymerization modulator technology specially designed for bulk-filling technique. By changing the initiator in these composite resins it has become possible to place composite resin at thicknesses greater than 4 mm, which results in significantly shorter chair times during the restorative procedures.^9^ It has also been shown that the depth of cure at these thicknesses is greater than that in nanofilled composite resins with the same thickness.^18^

Palin et al^19^ demonstrated that the microleakage of a Class V cavity restored with a silorane-based composite resin was not significantly different from that of a similar cavity restored with a conventional methacrylate-based composite. On the other hand, a number of studies have shown that silorane-based composite resins exhibit significantly lower shrinkage forces, less microleakage and better marginal adaptation than conventional methacrylate-based composite resins.^20,21^

EL-Damanhoury and Platt showed that bulk-filled composite resins have significantly less polymerization shrinkage compared to conventional posterior composite resins.^4^ However, according to Heintze et al,^22^ no significant differences were detected between the marginal quality of composite resin restorations placed in bulk and those placed in three increments.

At present, the chief concern about curing bulk-fill composite resins is the amount of polymerization shrinkage and the subsequent gap formation. Such shrinkage is more important at cervical margins of proximal boxes.^23^ Moorthy et al^24^ showed that the bulk-fill flowable RBC bases resulted in a significant decrease in cuspal deflection compared to a conventional RBC restored in an oblique incremental filling technique with no change in cervical microleakage.

The present study was undertaken to evaluate the effect of the type of light-curing unit and the location of the gingival margin, i.e. enamel and dentinal margins, on gap formation in Cl II cavities restored with bulk-fill silorane- and methacrylate-based composite resins. The null hypothesis of the study stated that the type of composite resin, light-curing unit and gingival margin location has no effect on gap formation.

## Methods

Sixty third molars without caries and defects were selected after surgical extraction due to impaction or semi-impaction. The teeth were cleansed with scaling curettes and immersed in 0.5% chloramine solution (Kemika, Zagreb, Croatia) for one month. Then two standard Cl II MO and DO cavities were prepared in the teeth using a #4 round diamond and a #245 fissure bur (Mani Inc, Utsuno-miya, Tochigi, Japan) in a high-speed handpiece (NSK, Tochigi-Ken, Japan) under air and water spray. One new bur was used for each eight cavities. The cavities measured 2.0 mm in the occlusal isthmus depth, 5.0 mm in the buccolingual width of the proximal box in the occlusal area, 5.5 mm in the gingival area, 2.5 mm in the depth of the axial wall in the occlusal area and 1.5 mm in the gingival area; in addition, the box height was 1 mm occlusal to the CEJ on the mesial aspect and 1 mm apical to the CEJ in the distal aspect. The dimensions mentioned above were confirmed with the use of a #15 UNC periodontal probe. Bevels were not placed at the cavosurface margins.

The teeth were randomly assigned to 4 groups with 15 teeth or 30 cavities in each group. The mesial and distal cavities of all the samples were etched for 15 seconds, rinsed and dried with cotton pellets.

**Group A:** Filtek P90 composite resin (FS) and quartz-tungsten-halogen (QTH) light-curing unit

First the P90 system self-etch adhesive primer (3M ESPE, St. Paul, MN, USA) was applied to the cavity walls with a microbrush, following manufacturer’s instructions, followed by the application and curing of P90 system adhesive bond for 10 seconds (3M ESPE, St. Paul, MN, USA). Then the cavities were restored with Filtek P90 composite resin (3M ESPE, St. Paul, MN, USA) using the bulk-fill technique and light-cured with the QTH light-curing unit.

**Group B:** Filtek P90 composite resin and light emitting diode (LED) light-curing unit

The cavities were restored in a manner similar to that in group A except that an LED light-curing unit was used in this group.

**Group C:** X-tra Fil composite resin and QTH light-curing unit

First the Clearfil SE Bond (Kuraray, Osaka, Japan) self-etch system was applied to the cavity walls with a microbrush, following manufacturer’s instructions, and cured for 10 seconds. Then the cavities were restored with X-tra Fil composite resin (VOCO Gmbh, Cuxhaven, Germany) using the bulk-fill technique and light-cured with the QTH light curing unit.

**Group D:** X-tra Fil composite resin and LED light-curing unit

The cavities were restored in a manner similar to that in group C except that an LED light-cuing unit was used in this group.

QTH Astralis 7 (Ivoclar, Vivadent, Lichtenstein) and LED Demetron A2 (Kerr, Donbury, Italy) were used for light-curing of composite resins at a light intensity of 1000 mW/cm^2^ for 20 and 10 seconds, respectively. Table 1 presents the particulars of the materials used in the present study.

**Table 1:** Material used and their composition

Material	Type	Composition	Batch No	Manufacturer	Application mode
Filtek P90 (silorane)	Matrix expanding Composite	Silane treated quartz ;yttrium trifluride; Bis-3,4-Epoxycyclohexyl-phenyl-Methysilane ;3,4-Epoxycyclohexylcyclo-Polymethylsiloxane	N384451	3M ESPE, St Paul, MN, USA	Bulk fill technique
X-tra Fil	Bulk-fill restorative	MMA, Bis EMA, Inorganic fillers	1147096	Voco, Cuxhaven, Germany	Bulk fill technique
Silorane adhesive		Primer: phosphorylated methacrylates, vitrebond copolymer, bis-GMA, HEMA, water, ethanol, silane-treated silica filler, initiators, stabilizers Bond: hydrophobic dimethacrylate, phosphorylated methacrylates, TEGDMA, silane treated silica filler, initiators, stabilizers	N213019 N213052	3M ESPE, St Paul, MN, USA	Apply primer to tooth surface ;gently air dry the surface and light cure for 10 s; apply bond ;gently air dry and light cure for 10s
Clearfil SE Bond		Primer: MDP, HEMA, hydrophilic dimethacrylate, photoinitiator, water Bond:10-MDP,bis GMA,HEMA hydrophilic dimethacrylate, microfiller, photoinitiator	1039AA 1550AA	Kurary Medical Inc, Okayama, Japan	Apply primer to tooth surface; air dry for 10s;applay bond; dry for 10 s and light cure for 10s.
Abbreviations: MMA: Methyl methacrylate, Bis EMA: Bisphenol A polyetheylene glycol diether dimethacrylate, Bis GMA: Bisphenol-A-glycidyl methacrylate, UDMA: Urethane dimethacrylate, TEGDMA: Triethyleneglycol dimethacrylate, HEMA:2-hydroxyethyl methacrylate, MDP: Methacryloxydecyl dihydrogen phosphate	Abbreviations: MMA: Methyl methacrylate, Bis EMA: Bisphenol A polyetheylene glycol diether dimethacrylate, Bis GMA: Bisphenol-A-glycidyl methacrylate, UDMA: Urethane dimethacrylate, TEGDMA: Triethyleneglycol dimethacrylate, HEMA:2-hydroxyethyl methacrylate, MDP: Methacryloxydecyl dihydrogen phosphate	Abbreviations: MMA: Methyl methacrylate, Bis EMA: Bisphenol A polyetheylene glycol diether dimethacrylate, Bis GMA: Bisphenol-A-glycidyl methacrylate, UDMA: Urethane dimethacrylate, TEGDMA: Triethyleneglycol dimethacrylate, HEMA:2-hydroxyethyl methacrylate, MDP: Methacryloxydecyl dihydrogen phosphate	Abbreviations: MMA: Methyl methacrylate, Bis EMA: Bisphenol A polyetheylene glycol diether dimethacrylate, Bis GMA: Bisphenol-A-glycidyl methacrylate, UDMA: Urethane dimethacrylate, TEGDMA: Triethyleneglycol dimethacrylate, HEMA:2-hydroxyethyl methacrylate, MDP: Methacryloxydecyl dihydrogen phosphate	Abbreviations: MMA: Methyl methacrylate, Bis EMA: Bisphenol A polyetheylene glycol diether dimethacrylate, Bis GMA: Bisphenol-A-glycidyl methacrylate, UDMA: Urethane dimethacrylate, TEGDMA: Triethyleneglycol dimethacrylate, HEMA:2-hydroxyethyl methacrylate, MDP: Methacryloxydecyl dihydrogen phosphate	Abbreviations: MMA: Methyl methacrylate, Bis EMA: Bisphenol A polyetheylene glycol diether dimethacrylate, Bis GMA: Bisphenol-A-glycidyl methacrylate, UDMA: Urethane dimethacrylate, TEGDMA: Triethyleneglycol dimethacrylate, HEMA:2-hydroxyethyl methacrylate, MDP: Methacryloxydecyl dihydrogen phosphate

All the restorations were finished with finishing disks containing aluminum oxide from coarse to fine (Sof-Lex TM, 3M ESPE, St. Paul, USA) based on manufacturer’s instructions. The samples were incubated (Irankhodsaz Co., Tehran, Iran) in distilled water at 37°C for 1 week, followed by thermocycling under standard conditions (5/55±5°C, 500 cycles).

**Figure 1:**
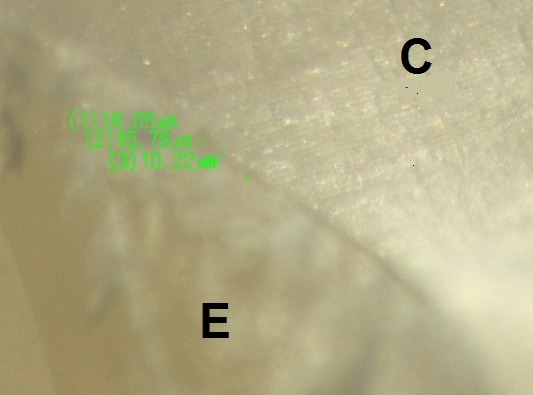
Sample micrograph of gap measurements (E: enamel, C: composite).

Subsequently, the teeth were bisected mesiodistally using diamond disks (Diamond GmbH, D&Z, Berlin, Germany) and marginal adaptation was evaluated under a stereomicroscope (SMZ 800, Nikon, Tokyo, Japan) at ×60. Selected areas underwent a digital radiographic procedure with the use of a digital imaging system (DS Camera Control Unit, DS-LZ Ver. 4.4) and transferred to a computer to measure gaps. A software program (DS Camera Control Unit, DS-LZ Ver. 4.4) was used to measure the width of the interfacial gaps at 3 points and their mean was recorded in µm as the mean gap size (Figure 1). Data were analyzed with SPSS 20, using the General Linear Model (GLM) multivariate variance analysis. Bonferroni post hoc tests were used for pair-wise comparisons. Statistical significance was set at P<0.05.

## Results

Table 2 presents the mean gap sizes (µm) in the study groups.

**Table 2:** Mean gap measurements in the study groups (µm)

**Composite resin type**	**Margin location**	**Light-curing unit**	
		**LED**	**QTH**
**X-tra Fil**	Enamel	7.07^A^	7.21^A^
	Dentin	11.46^b^	12.07^b^
**Filtek P90**	Enamel	7.74^C^	7.96^C^
	Dentin	13.02^d^	13.28^d^

**Figure 2:**
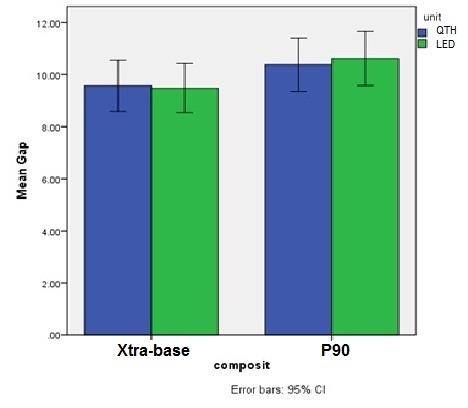
The error bars of the cumulative effect of composite resin‒light curing unit.

**Figure 3:**
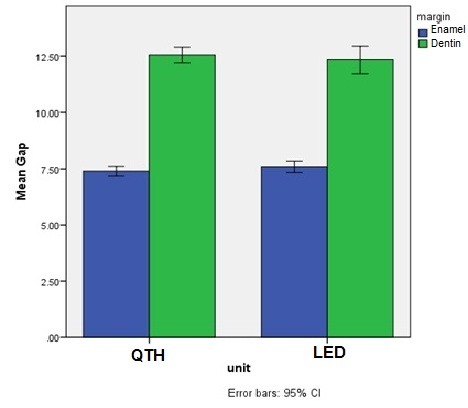
The error bars of the cumulative effect of margin location‒light curing unit.

**Figure 4:**
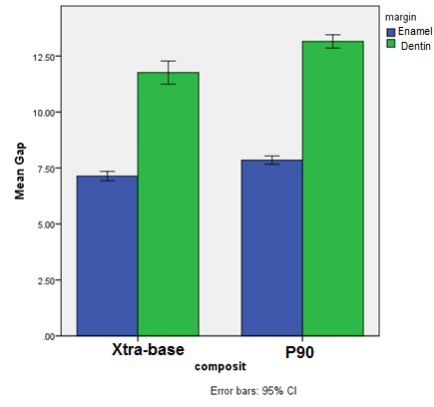
The error bars of the cumulative effect of composite resin‒margin location.

The results of GLM analysis showed significant differences between the two composite resin types (P<0.001) and between the two margin types (P<0.001); however, the differences between the two light-curing units were not significant (P=0.97). Evaluation of the interactive effects of variables with the use of GLM analysis showed no significant interactive effect of composite resin type‒light-curing unit (P=0.14) (Figure 2), margin location‒light-curing unit (P=0.27) (Figure 3) and composite resin‒light-curing unit‒margin type (P=0.22). However, the interactive effect of composite resin‒margin was statistically significant (P=0.04) (Figure 4).

Two-by-two comparisons using Bonferroni post hoc test showed that the gaps with the use of silorane-based composite resin were larger than those with the use of X-tra Fil composite resin (P<0.001), with larger gaps at dentinal margins compared to the enamel margins (P<0.001). X-tra Fil composite resin exhibited smaller gaps at both enamel and dental margins with both LED and QTH light-curing units compared to the silorane-based composite resin (P<0.001).

## Discussion

The results showed that the composite resin type affected gap formation, and the size of gaps with the X-tra Fil composite resin was smaller than that with the silorane-based composite resin at both enamel and dentin margins, irrespective of the type of the light-curing unit.

The differences between dissimilar letters are significant (P<0.05).

Contrary to incremental insertion technique in which composite resins are placed in 2-mm-thick layers in the cavity to decrease polymerization shrinkage and achieve proper depth of cure,^25,26^ in the bulk-fill technique composite resin is placed in 4‒6-mm layers in the cavity, saving considerable time during restorative procedures due to the restoration of the cavity in one step.

The longevity of a composite resin restoration depends on several factors, including the composite‒cavity interfacial seal.^27^ In cases in which there is inadequate bonding to tooth structures, forces resulting from polymerization shrinkage might give rise to gap formation at cavity wall‒restorative material interface.^28^ Composite resin marginal integrity might be affected by various factors, including the cavity size, the angle at which enamel prisms and dentinal tubules are cut based on their location, the procedure in which dental hard tissues are conditioned, the layering protocol and the polymerization technique used, etc.^29^ Therefore in the present study, the effects of composite resin type, light-curing unit type and the gingival margin location on gap formation were evaluated in Cl II restorations with the use of the bulk-fill restorative technique.

Similarly, Van Ende et al^30^ demonstrated that the type of the bulk filling composite has a great effect on bonding efficacy. They postulated that differences in bond strength between composites can be attributed to differences in shrinkage stress. Shrinkage stress is not a material’s property, but is inherent to the compliance and C-factor. However, in the present study standard class II cavities with similar dimensions were prepared; therefore, the C-factor was similar in all the samples. Therefore, it appears that differences in the polymerization mechanisms are responsible for differences in gap formation between these two different types of composite resin. Bulk-fill composite resins, such as X-tra Fil, have been designed for placement in 4-mm or thicker increments and it has been shown that the amount of polymerization shrinkage is low and acceptable at this thickness.

Bulk-fill composite resins exhibit a greater depth of cure and lower polymerization shrinkage compared to conventional composite resins, which is attributed to the chemical embedding of ‘polymerization modulators’ in the polymerizable resin backbone of the resin monomer, delaying the gel point. In the pre-gel phase, the polymer chains are very flexible, allowing the material to flow from the free surface of the cavity. The developing polymer’s viscosity is still low; therefore, shrinkage stress is compensated by the plastic flow during the pre-gelation phase. As a result, the internal stresses within the material are relaxed.^31^ The time at which the material can no longer compensate the polymerization contraction (the time until gelation) determines the final tensions in the material. However, compared to conventional methacrylate-based materials, this type of composite resins behaves differently, with a delayed gelation point, statistically similar to silorane-based composite resins only.^32^

The slow polymerization of the resin is another mechanism that compensates stresses in composite resins; it increases the resin’s flow capacity.^33^ Based on previous reports, silorane composite resins have a slower initiation of polymerization reaction and more time is necessary for formation of cations.^19,34^ Compared to conventional methacrylate-based composite resins, the shrinkage stress in silorane composite resins is significantly lower and the gelation time is significantly longer.

Furthermore, Van Ende et al^35^ reported that bulk-filling with Filtek Silorane significantly decreases the bond strength, suggesting that factors other than polymerization shrinkage may influence its bond strength. Only when FS was used in three layers and cured separately, an equally high µTBS was measured compared to conventional and low-shrinkage methacrylate-based composite resins. When FS was applied in bulk, a significantly lower bond strength was recorded. The higher bond strength of FS upon layering might be attributed to more effective polymerization of thinner layers. Additionally, several studies have reported lower hardness at the bottom of the cavity with the use of silorane composite resins in bulk.^36,37^ In this regard, based on a recent report, there was a significant effect of energy dose and longer curing time on the hardness and bond strength with the use of silorane composite resins, which was not manifest for the methacrylate-based composite resins, irrespective of the curing depth.^35,38^

It has been reported that the shrinkage stresses of a material depend on its elastic modulus. The stresses at interfacial areas during the setting shrinkage of a composite resin are positively correlated with the rigidity of the material based on in vitro studies.^39^ The elastic modulus, rigidity and mechanical properties of polymers are‏ associated with cross-linking density of the polymer network and‏ the total conversion rate.^40^ Yamasaki et al^41^ demonstrated that FS exhibited the highest cross-link density, probably‏ due to the presence of di- and tetra-functional molecules‏ in its composition, which justifies its high modulus and flexural‏ strength compared to methacrylates, as reported by Weinmann et al.^15^ This agrees with the results observed‏ in this study, in which FS containing multifunctional monomers‏ with the highest cross-link density and greater rigidity and less flexibility due to its higher elastic modulus showed greater gaps in its interface.

In addition, in the current study, two different types of light-cuing units, i.e. LED and QTH, were used for polymerization of composite resin samples. These types of light-curing units were selected due to their popularity and more widespread use by clinicians compared to other light-curing units. However, no differences were observed in gap formation with the use of composite resins polymerized in a similar manner with the use of different types of light-curing units, which coincides with the results of some previous studies.^42,43^ Similarly, Lee et al^44^ showed that the amount of polymerization shrinkage is a factor of the type and composition of composite resin, rather than the type of light-curing unit.

Zakavi et al^45^ reported no statistically significant differences in microleakage at enamel margins between LED and QTH light-curing units; however, significant differences were detected at dentin margins, with LED units exhibiting better performance at dentin margins, which was attributed to the higher consistency between the radiation spectrum of LED units and the absorption spectrum of camphorquinone which is the photoinitiator in conventional composite resins. In contrast, Casseli et al^46^ showed that the type of the light-curing unit affected gap formation at both the enamel and dentin interfacial areas. The differences might be attributed to differences in adhesive systems and composites used.

Another important result of this study was the fact that the mean gap sizes with the use of both composite resins were smaller at enamel margins in comparison to dentinal margins, consistent with the results of similar previous studies.^45,47,48^ Since enamel has a homogeneous structure, bonding to enamel is reliable and is achieved with ease; however, it is much more difficult to achieve a favorable bond with dentin, which is attributed to factors such as the non-homogeneous structure of dentin, flow of the dentinal tubular fluid toward the external surface and lower mineral content of dentin compared to enamel.^49^ Previous studies have reported similar findings with the use of different types of composite resins, including conventional,^50^ packable^50^ and nano and silorane composite resins.^48^

Under the limitations of this in vitro study, with no use of different types of composite resins and curing techniques, it is suggested that in future studies, gaps be measured under a scanning electron microscope (SEM), different bulk-fill composite resins, and other light-curing techniques, including soft start and ramped curing modes.

## Conclusion

It was concluded under the limitations of this study that:

1. The type of the light-curing unit did not affect gap formation.
2. The type of the composite resin affected gap formation, with smaller gaps with the use of X-tra Fil composite resin compared to the silorane-base composite resin.
3. There were smaller gaps at enamel margins compared to dentinal margins, irrespective of the type of light-curing unit and the type of the composite resin used.

## Acknowledgement

The authors extend their appreciation to the Office of the Vice Chancellor for Research and Dental and Periodontal Research Center Tabriz University of Medical Sciences, for the financial support of this research. This article was derived from an MSc degree thesis in operative dentistry (No.210/T) in Tabriz Faculty of Dentistry. Furthermore, the authors kindly appreciate Dr Morteza Ghojazade for statistical analysis of data and Dr Majid Abdolrahimi for translation of the article into English.

## Authors’ contributions

This study was planned by SSO and MB. The literature review was performed by MB, SSO, EJN and AAA. NG and ASO performed the experiments. The statistical analyses and interpretation of data were carried out by ENJ and AAA. MB and NG and ASO drafted the manuscript. All the authors critically revised the manuscript for intellectual content. All the authors have read and approved the final manuscript.

## Funding

The authors would like to thank the Office of the Vice Chancellor for Research and Dental and Periodontal Research Center, Tabriz University of Medical Sciences, for the financial support of this study.

## Competing interests

The authors declare no competing interests with regards to the authorship and/or publication of this article.

## Ethics approval

The study protocol was approved by the Research Ethics Committee of Tabriz University of Medical Sciences.
